# A community Milk Share initiative promoting health equity through Black community leadership

**DOI:** 10.3389/fpubh.2025.1589089

**Published:** 2025-07-04

**Authors:** Elizabeth Mollard, Cydney Gaines, Jillian Boldt, Jordan Hardesty, Clara Fynbu Eggert, Ebony Reddick, Tina Goodwin

**Affiliations:** ^1^University of Nebraska Medical Center, Omaha, NE, United States; ^2^Maternal Wellness Program, The Malone Center, Lincoln, NE, United States; ^3^Frontier Pediatric Care, Lincoln, NE, United States; ^4^Women's and Men's Health, Every Woman Matters, Nebraska Department of Health and Human Services, Lincoln, NE, United States

**Keywords:** breastfeeding disparities, milk sharing, community led initiative, black maternal health, breastfeeding, health equity, Milk bank for donated mother’s milk

## Abstract

Persistent racial disparities in breastfeeding reflect broader systemic inequities that disproportionately impact Black families. In response to barriers such as limited access to culturally competent lactation support, biased hospital practices, and financial constraints, Black-led, community-driven initiatives have emerged to improve breastfeeding success and infant nutrition equity. This community case study describes the development and implementation of the Malone Maternal Wellness Milk Share program, a Black-led initiative providing structured, community-based milk sharing to ensure equitable access to human milk. The program addresses systemic barriers by integrating rigorous donor screening protocols, informed consent processes, and culturally responsive lactation support while maintaining accessibility for families who might not otherwise have access to traditional milk banks. While created to address racial disparities, the Milk Share program is intentionally inclusive, welcoming all families in need regardless of race, ethnicity, or background. A Community Advisory Board guides the program, ensuring it remains aligned with both public health safety standards and the needs of Black families. By embedding donor human milk access within a broader framework of maternal health support, the Milk Share program demonstrates the effectiveness of community-led solutions in mitigating breastfeeding disparities and promoting infant health. The findings from this initiative underscore the need for healthcare systems and policymakers to recognize and support community-driven interventions as critical components of maternal and infant health equity efforts.

## Introduction

Breastfeeding is widely recognized for its substantial benefits to both maternal and infant health. However, persistent racial disparities in breastfeeding initiation and duration reflect broader systemic inequities (1, 2). Black women in the United States experience lower breastfeeding rates due to multiple structural barriers, including limited access to culturally competent lactation support, biased hospital practices, and inadequate healthcare provider guidance (3, 4). Disparities in early breastfeeding experiences, such as skin-to-skin contact and timely initiation, are consistently promoted for white women but not for Black mothers (5). Workplace constraints further exacerbate breastfeeding disparities, as Black women are disproportionately employed in positions with minimal paid maternity leave, inadequate lactation accommodations, and rigid schedules that impede the ability to pump or breastfeed (3, 6–8).

Historical factors also shape contemporary breastfeeding disparities. The legacy of slavery, in which Black women were forced to serve as wet nurses for white families, disrupted maternal–infant bonds and contributed to enduring stigmas surrounding breastfeeding in some Black communities (9, 10). Additionally, decades of formula marketing targeted at communities of color, coupled with a healthcare system that has historically undervalued breastfeeding education and support for Black mothers, have further entrenched disparities (3, 11).

Addressing these inequities requires structural interventions including policies that expand paid parental leave, enforce workplace protections for lactating employees, and increase access to culturally congruent community-based lactation support. While such systemic changes are critical, they do not meet the immediate needs of Black families navigating the daily challenges of breastfeeding within an inequitable healthcare system. In response, community-driven solutions have emerged, leveraging cultural strengths and collective support to promote breastfeeding success among Black families. This case study is grounded in a health equity framework, recognizing that interventions must address structural racism and resource disparities that shape breastfeeding outcomes.

### The value of human milk and the consequences of breastfeeding disparities

Human milk is the optimal source of infant nutrition, providing essential biological and immunological protections that support survival, growth, and development. The unique composition of breast milk includes antibodies, growth factors, and microbiota that play a critical role in reducing infant morbidity and mortality, protecting against infectious diseases, and promoting long-term health benefits (12, 13). These protective effects extend beyond infancy, influencing immune function, metabolic health, and neurodevelopment well into adulthood (14). Beyond individual benefits, breastfeeding contributes to broader public health improvements by reducing the incidence of chronic diseases and lowering healthcare costs associated with formula feeding (14).

The consequences of breastfeeding disparities among Black infants are profound. Breastfeeding significantly reduces the risk of sudden infant death syndrome (SIDS), metabolic disorders, asthma, and other chronic conditions (15). Yet, Black infants who already experience higher rates of these conditions are the least likely to receive human milk’s protective benefits (15). Additionally, Black infants face increased rates of preterm birth, leading to higher risks of complications such as necrotizing enterocolitis (NEC), sepsis, and respiratory issues, conditions for which human milk substantially decreases morbidity and mortality (15, 16).

Systemic barriers that restrict access to breastfeeding support and donor human milk disproportionately expose Black infants to preventable health disparities (3, 16, 17). Infant nutrition insecurity is heightened in communities where structural barriers to breastfeeding have led to a reliance on formula feeding. This vulnerability was starkly exposed during the 2022 formula shortage, caused by supply chain disruptions and a major recall from bacterial contamination concerns at a key production facility, resulting in widespread formula scarcity (11, 18). Black families, already disadvantaged by breastfeeding disparities and targeted by aggressive formula marketing, experienced disproportionate impacts during this crisis, further exacerbating nutritional inequities in Black communities (11, 18). The shortage highlighted the fragility of formula-dependent systems and reinforced the urgency of equitable lactation support and accessible human milk alternatives.

While intended to support infant health, human milk banks, and donation programs often exacerbate disparities by reinforcing systemic inequities in access and affordability (19). Research shows that donor human milk is disproportionately distributed to wealthier, predominantly white families (19). At the same time, Black mothers, who experience higher rates of preterm birth and infant mortality, face barriers to obtaining donor human milk (19). Milk banks require physician prescriptions and charge high fees, making donor human milk inaccessible for Black families with limited healthcare access or financial resources (19, 20). Moreover, Black women are more likely to donate milk than to receive it, creating a cycle reminiscent of slavery, when Black women provided milk for white infants while their own children remained underserved (19). The current human milk banking system depends on Black women’s generosity to support others, even as Black infants face limited access to donor human milk (21). This dynamic reflects broader racial inequities in maternal and infant healthcare, where Black mothers and infants encounter systemic barriers to accessing life-saving interventions, essential care, and vital resources (22). Without policies that ensure equitable access, milk banking risks deepening racial health disparities rather than addressing them.

### Black-led, community-driven solutions to human milk disparities

Black-led, community-driven initiatives have played a critical role in addressing breastfeeding disparities by providing culturally relevant, accessible, and holistic support to Black families (23, 24). These initiatives counteract systemic barriers such as medical racism, economic constraints, lack of culturally competent lactation care, and limited social support by centering the lived experiences of Black parents and integrating traditional models of communal care (3, 4, 9).

One key feature of Black-led breastfeeding initiatives is the emphasis on culturally competent lactation support. Community programs prioritize training Black lactation professionals, such as Certified Lactation Counselors (CLCs) and International Board-Certified Lactation Consultants (IBCLCs), to provide evidence-based care within a culturally affirming framework (3, 4). Research indicates that peer counseling programs led by individuals from the same racial, ethnic, or cultural background significantly increase breastfeeding initiation and exclusivity rates, as they offer not only practical support but also emotional validation and culturally relevant guidance (23, 25).

Another cornerstone of Black-led breastfeeding interventions is the integration of wraparound services that address social determinants of health impacting lactation success. Economic instability, food insecurity, and lack of paid parental leave disproportionately affect Black mothers, making it more difficult to initiate and sustain breastfeeding (3, 26). Community organizations have responded by incorporating lactation support into broader maternal health initiatives that provide financial assistance, nutritional support, mental health resources, and postpartum care.

Social support networks also play a crucial role in improving breastfeeding outcomes. Historically, Black communities have relied on intergenerational knowledge-sharing and communal caregiving to support infant feeding, but medicalized childbirth practices and structural racism have disrupted these traditions (27). Black-led breastfeeding groups have created peer mentoring networks and support circles to restore these models, providing a space where Black mothers can share experiences, receive encouragement, and normalize breastfeeding within their communities (28, 29). Research has demonstrated that Black mothers with access to culturally congruent breastfeeding support groups are more likely to initiate and sustain breastfeeding compared to those who rely solely on traditional medical providers (30).

These community-driven solutions highlight the power of Black maternal health leadership in transforming breastfeeding outcomes and advancing health equity. By shifting the focus from deficit-based narratives to asset-based approaches, Black-led initiatives improve breastfeeding rates and reassert the importance of community autonomy, self-determination, and culturally centered care in maternal health.

## Context

Malone is an African American Community Center in Lincoln, Nebraska, serving Lincoln, Omaha, and rural Nebraska areas. For the past 70 years, Malone has created and facilitated programs to combat the racial disparities and inequities in justice, healthcare, and social systems. They have served as a cornerstone for innovative educational, cultural, advocacy, and health and welfare programs. The Malone Maternal Wellness (MMW) Program is a Black-led community initiative that supports maternal and infant health by providing culturally responsive care, education, and resources to Black families and other underserved populations. The following sections will explore the development, implementation, and impact of the MMW’s Milk Share initiative, highlighting how Black-led community innovation can overcome breastfeeding disparities care by prioritizing equity, empowerment, and cultural resilience.

## Details

### Developing the community Milk Share program

Milk sharing, or the practice of one lactating person providing human milk to another’s infant, has long served as a communal strategy to ensure infant nutrition (31). While informal milk sharing has long existed within close-knit communities and extended families across the world, modern programs aim to bring structure and safety to the practice while maintaining accessibility. The Milk Share program at MMW was developed as a community-based initiative to provide equitable access to donor human milk for families who might not otherwise have access to traditional milk banks. Although led by Black community leaders to address racial breastfeeding disparities, the Milk Share initiative intentionally welcomes participation from all members of the broader community, enabling anyone to donate or receive milk regardless of race, ethnicity, and/or socioeconomic background. The program addresses logistical and ethical concerns by combining rigorous safety protocols with a culturally responsive and inclusive framework while preserving the communal spirit of milk sharing.

### Recognizing the need

The MMW Milk Share program was created in response to a need identified through close interactions between MMW’s lactation providers and their clients. Although the center already offered extensive lactation support, including in-home visits, 24/7 virtual support, and breastfeeding support groups for mothers and fathers, the team noticed that some women struggled with low milk supply while others had an oversupply. Additionally, MMW families relying on formula faced challenges due to the 2022 formula shortages. The stigma and perceived risks associated with alternative infant feeding options, such as homemade formula or whole milk, further complicated the situation.

Conventional milk banks were not a feasible option for families due to their high cost, prescription-only requirements, and limited availability, typically for inpatient preterm or medically fragile infants. Informal milk-sharing networks, often facilitated online, lacked standardized health screenings for donor health, drug use, or food sensitivities, posing significant health concerns (31, 32). Recognizing these challenges, the necessity of a structured, community-driven milk-sharing program became evident.

### Considerations of risks of milk sharing

The American Academy of Pediatrics (AAP) and the Food and Drug Administration (FDA) discourage informal milk sharing due to safety concerns (32, 33). The AAP warns that non-pasteurized donor human milk and other forms of informal milk sharing, such as direct exchange or internet-based transactions, pose risks of bacterial or viral contamination and potential exposure to medications, drugs, or herbs (33). Similarly, the FDA advises against obtaining human milk from online sources, citing studies that have found bacterial contamination in some milk samples obtained through internet-based sharing (32). Instead, these organizations advocate for using pasteurized donor human milk from established milk banks to ensure safety for infants.

Despite these recommendations, families faced difficulty accessing formula, banked milk was inaccessible and unaffordable, and professional organizations recommended against making homemade formula. The community’s need for human milk demanded an urgent and innovative solution.

### Model identification and program framework

Existing nonprofit milk-sharing programs were evaluated to develop a sustainable and safe milk-sharing model. Although these programs do not operate as formal milk banks, they provide a structured alternative to informal online milk sharing. A non-profit model program that required participants to undergo comprehensive medical and blood screening for transmissible diseases was selected as a foundational framework. This program provided recipients with clear instructions on self-pasteurization to enhance milk safety. This approach balanced accessibility with health safeguards and informed the structural development of the Milk Share initiative.

Unlike commercial donor human milk programs, which often require prescriptions and charge high fees, the Milk Share program prioritized equity and inclusivity, ensuring that all families, regardless of race, income, or insurance status, had access to donor human milk. While it specifically addressed barriers faced by Black and marginalized communities, the program remained open to anyone in need. The development process involved engaging key stakeholders, establishing safety and screening protocols, and creating an infrastructure for milk collection, storage, and distribution.

### Establishing the community advisory board

A crucial component of the program’s development was the formation of a Community Advisory Board (CAB) to ensure that the Milk Share remained community-centered and evidence-based. The CAB comprised diverse stakeholders, including a pediatrician, nurse practitioner, nurse-midwife, lactation specialized pharmacist, lactation consultants, maternal health advocates, community leaders, and parents with lived experience in breastfeeding and milk sharing. By integrating the voices of those directly affected by breastfeeding disparities, the CAB played a critical role in shaping program policies, safety measures, and outreach strategies.

**Figure 1:**
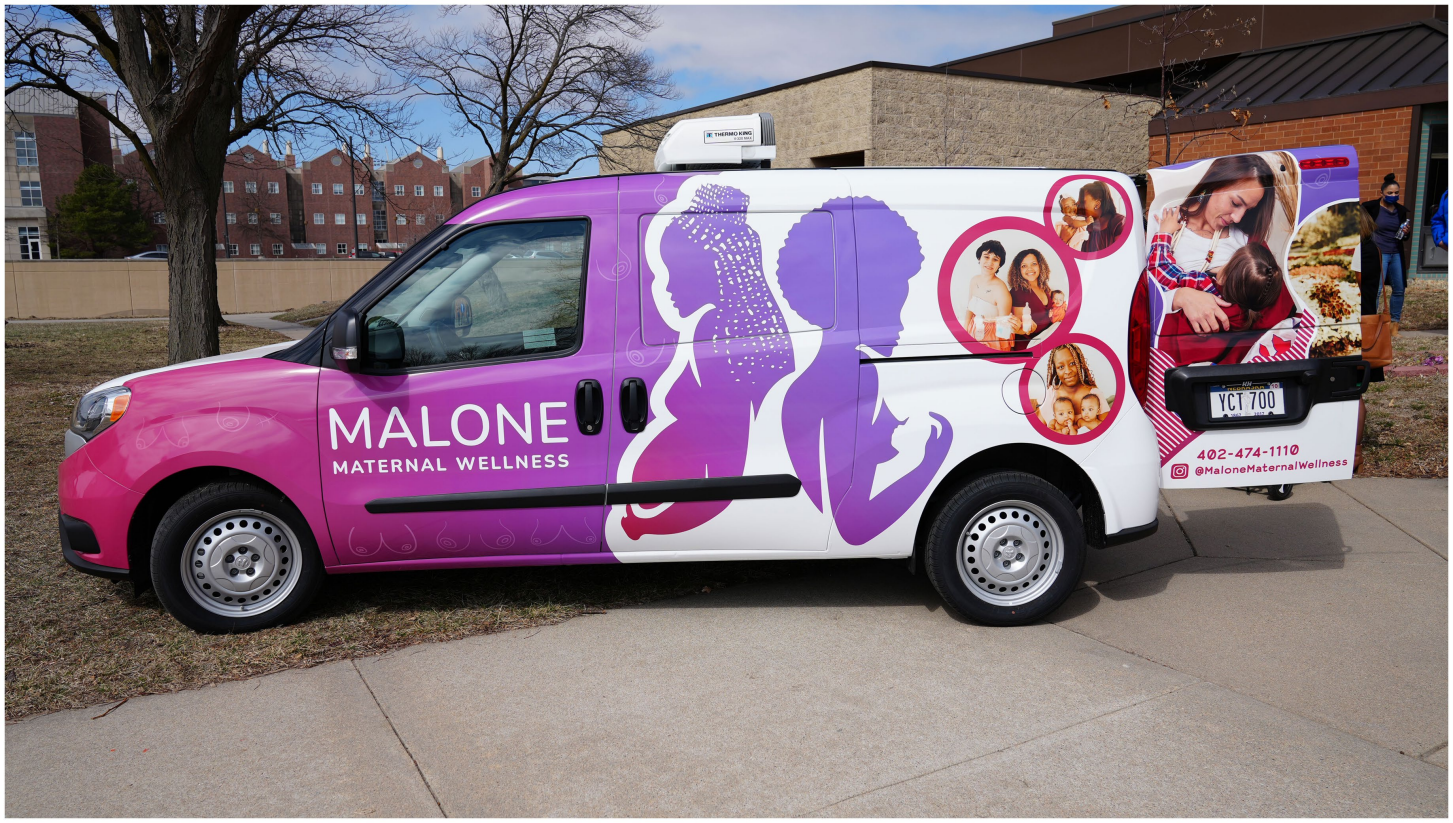
The refrigerated Malone Milk Mobile enables the safe transport of human milk to and from community sites, reducing logistical barriers for families and improving access to donor and shared milk resources. Reproduced from: https://malonecenter.org/maternal-wellness/, Malone Center.

The CAB convened regularly to address emerging challenges, assess program outcomes, and refine operational protocols. This collaborative structure ensures that the Milk Share remains responsive to the community’s needs while maintaining credibility among healthcare professionals and public health officials. Additionally, the CAB facilitated partnerships with local healthcare providers and advocacy organizations, strengthening the program’s sustainability and reach. Through their involvement with the community board, the program secured funding from a Medicaid-managed care organization serving the local population and received support from Frontier Pediatric Care, which provided discounted lab testing services (Figure 1).

### Structured donor screening and approval process

The success of the MMW Milk Share program relies on the development of a structured, community-driven approach that ensures both accessibility and safety. Potential milk donors undergo comprehensive health screenings, including a detailed medical history assessment, laboratory testing for transmissible diseases such as Human Immunodeficiency Virus (HIV), hepatitis B and C, syphilis, and Human T-cell lymphotropic Viruses (HTLV-1 and HTLV-2), and a five-panel drugs screen (testing for marijuana, cocaine, opiates, amphetamines, and phencyclidine [PCP]) similar to the process involved with participation in formal milk banking (34). Additionally, donors are screened for medication use, dietary restrictions, and substance exposure to ensure the quality and safety of donated milk.

Milk Share recipients are provided comprehensive details about the donor screening process to maintain transparency and aid in informed decision-making. While the program does not require donors to pasteurize their milk before distribution, recipients are educated on at-home flash-heating methods, a widely recognized strategy for reducing potential pathogen exposure without significantly diminishing the nutritional and immunological properties of human milk (35).

### Safe handling and storage protocols

Proper handling and storage of donated milk are critical components of the program’s safety framework. The program developed detailed guidelines based on best practices from established human milk banks and lactation organizations. Donors are provided with sterile collection and storage containers and recommendations on proper milk expression techniques, refrigeration, and freezing methods. The program also facilitated regular community workshops on safe milk handling, equipping families with the knowledge and skills to minimize contamination risks.

A new refrigerated breast milk-sharing vehicle, affectionately named the “Milk Mobile,” was funded by a Medicaid Managed Care organization to facilitate the collection and distribution of donated human milk (Figure 1). The Milk Mobile enables Malone staff to transport donor human milk directly from donor families to recipients within a two-hour radius of Malone, reducing logistical barriers. To ensure equitable distribution, priority is given to infants under 6 months of age, particularly those who were preterm, medically fragile, or had limited access to alternative feeding options.

### Community-based education and support

Recognizing that milk sharing is not solely about nutrition but also about fostering a supportive network, the MMW incorporated robust community education efforts into the program. Families engaging in milk sharing are invited to participate in lactation support groups, peer mentoring programs, and maternal health education sessions. These initiatives provide culturally affirming guidance while reinforcing traditional models of communal care, addressing both the emotional and logistical challenges of breastfeeding and milk sharing.

A key strength of the program is its emphasis on informed decision-making. Educational materials were developed in collaboration with attorneys, lactation consultants, a pharmacist and pediatric and maternal healthcare providers to ensure participants had access to evidence-based information about the benefits and potential risks of milk sharing. Parents who chose to participate are required to complete an informed consent process, which outlines considerations regarding donor screening, safe handling, and storage practices.

### Addressing ethical and logistical challenges

While the Milk Share program has successfully increased access to human milk within the community, its implementation was challenging. Concerns regarding liability, regulatory oversight, and medical endorsement initially created hesitancy among some community healthcare providers. In addition to working closely with an attorney on protocols and consents, the program’s advisory board engaged in active dialogue with pediatricians, neonatologists, and public health officials, emphasizing the program’s rigorous screening protocols and alignment with harm-reduction strategies.

Additionally, financial sustainability poses an ongoing challenge. Unlike formal milk banks, which often charge fees for processing and distributing milk, the Milk Share program is entirely free to participants. The program relies on private donations, grant funding, and community partnerships to support the Milk Share program. The establishment of the Milk Mobile, a dedicated vehicle for collecting and distributing donated milk, was a particularly innovative solution, reducing transportation issues, and concerns over the milk staying frozen during pickup and delivery, while expanding the program’s reach.

### Scaling and future directions

Following its initial success during the formula shortage, the Milk Share program has continued to evolve. Although not a formal research study, the program team documented implementation processes, collected anecdotal feedback, and tracked key metrics such as volume of milk donated, number of families served, and delivery frequency. Staff maintained internal records and notes from advisory board meetings, and informal interviews with participating families were used to guide program improvement. Program impact was evaluated based on both quantitative outputs and qualitative narratives of family satisfaction and engagement. In its first 2 years, the program has distributed 14,069.5 ounces of donor human milk to 41 families across Lincoln, Omaha, and surrounding areas, providing an estimated cost savings of over $88,000 for recipient families. The program now averages six to seven deliveries per week, continuing to fill critical gaps for families in need. Efforts are underway to expand its reach by increasing partnerships with healthcare institutions and integrating digital platforms for tracking milk donations and distributions. Additionally, the program continues to explore collaborations with local employers to advocate for workplace lactation support, ensuring that more parents can sustain breastfeeding while meeting their professional obligations.

Beyond ounces of milk distributed and families served, families reported high satisfaction with the program, citing the ease of access, trust in safety protocols, and emotional relief during a time of scarcity. Recipients frequently expressed gratitude for the culturally affirming support and guidance received. Lactation support group attendance also increased during the same period, suggesting enhanced community engagement around breastfeeding.

The ultimate goal of MMW and the Milk Share program is to combat the inequities and disparities in Black infant and maternal health by providing safe, culturally reflective perinatal care. The MMW Milk Share and breastfeeding support programs ensure that Black women have the lactation support they need and that Black infants receive optimal nutrition. By combining cultural strengths with evidence-based practices, the Milk Share program has emerged as a model for community-driven maternal and infant health initiatives. It underscores the importance of leveraging grassroots solutions to address breastfeeding disparities and demonstrates the transformative power of community-led innovations in improving health equity.

## Discussion

Black families, who have long faced systemic barriers to maternal and infant health resources, were among the hardest hit by the 2022 formula shortage. While supply chain disruptions and product recalls affected families across racial and economic lines, Black families were disproportionately impacted due to preexisting disparities in breastfeeding support and donor human milk access (11). The crisis exposed the fragility of infant feeding systems and the lack of sustainable alternatives to commercial formula. In response, many Black families turned to milk sharing, not only as a practical solution but as an act of community resilience.

The MMW Milk Share was an innovative, community driven solution to overcoming Black breastfeeding disparities and addressing infant nutrition security. Importantly, although driven by Black community leadership to specifically address systemic racial disparities, the Milk Share program intentionally provided equitable access to all community members, regardless of racial or cultural background. This inclusivity highlights that Black women, stereotypically portrayed as recipients of aid, are active leaders and providers of support challenging deficit-based narratives and underscoring their essential role in advancing community-wide health equity (36).

Health professionals warned against informal milk sharing or using homemade formulas due to contamination risks and inadequate nutrition (32, 33). While these concerns were valid, the emphasis on formula safety often overshadowed discussions about alternative feeding solutions, such as donor human milk, when provided with critical safety guards and informed consent. The MMW Milk Share emerged as a critical model, integrating rigorous screening protocols, safe handling procedures, and robust community support. The MMW Milk Share provided both immediate nutritional support and a pathway toward long-term maternal and infant health equity. Unlike large-scale, centralized donor human milk banks, which often impose financial and logistical barriers that are inaccessible to Black families, community-based milk sharing offers an accessible, culturally relevant solution.

Forming a CAB was pivotal in ensuring the program’s credibility and effectiveness. By engaging lactation professionals, healthcare providers, community stakeholders, and community members, the CAB played a crucial role in shaping policies, fostering trust, and aligning the program with the specific needs of Black families. This collaborative model strengthened the initiative’s legitimacy and demonstrated the power of community-led health interventions. Integrating peer mentoring and lactation support services further strengthened maternal–infant health outcomes, ensuring that milk sharing extended beyond nutrition to create a social and emotional support network.

The MMW Milk Share does more than address breastfeeding disparities, it represents a radical act of reclamation. Historically, Black women were forced to nourish white infants at the expense of their own children, perpetuating systemic inequities in maternal and infant health (10). In contrast, the milk sharing initiative discussed here reconfigures this dynamic. Black women are not only active participants but leaders in shaping the terms of milk sharing. By doing so, they assert agency over maternal and infant health in their communities, fostering a model of care that prioritizes empowerment, mutual aid, and health justice.

Furthermore, this initiative underscores the need for structural changes in how infant feeding is supported in the United States. While formula shortages were a wake-up call, they were not an anomaly. They revealed deeper systemic failures, including economic disparities, racial inequities in breastfeeding support, and the lack of investment in community-driven maternal health solutions. The MMW Milk Share program provides a compelling alternative, demonstrating the power of grassroots initiatives to fill gaps left by institutional failures.

To build a more resilient infant feeding infrastructure, policymakers and healthcare institutions must support and legitimize community-driven solutions. This includes expanding access to donor human milk, reducing financial barriers, and investing in culturally responsive breastfeeding support. By learning from initiatives like the one examined, healthcare systems can move toward an infant feeding model that is equitable, sustainable, and centered on community empowerment.

This model may be adapted to both urban and rural communities by maintaining core components such as structured donor screening, a community advisory board, and wraparound lactation support. Flexibility in delivery methods, staffing, and community partnerships allows the program to be tailored to local contexts. However, preserving community leadership, cultural congruence, and informed consent practices is critical to ensuring trust and sustainability.

## Conclusion

The MMW Milk Share program is not simply a response to breastfeeding disparities but a transformative model of Black maternal leadership and public health innovation. This initiative redefines how maternal and infant health care can be equitably delivered by centering cultural knowledge, historical resilience, and community collaboration.

Through this initiative, Black women reclaim control over infant feeding practices, shifting from historical oppression toward a future of empowerment and equity. Developing a structured, community-driven model that integrates safety, accessibility, and cultural relevance ensures that donor human milk is not just an option but a right for families facing systemic barriers to breastfeeding support.

Furthermore, the success of the Milk Share program demonstrates the potential for scaling community-led health interventions. Future initiatives should prioritize the role of community advisory boards, leverage peer support networks, and advocate for policy changes recognizing milk sharing as a legitimate and necessary component of maternal and infant health care.

The lessons from this program can inform broader public health strategies, demonstrating that the most effective solutions emerge from within the communities they serve. The MMW Milk Share program provides a powerful blueprint for equitable and sustainable maternal health solutions by shifting the paradigm from institutional dependence to community-led empowerment.

## Data Availability

The original contributions presented in the study are included in the article/supplementary material, further inquiries can be directed to the corresponding author.
